# Sonographic and Histological Morphometry of the Uterine Cervix—An Assessment of Laparoscopic and Other lntrafascial Hysterectomy Techniques

**DOI:** 10.1155/DTE.2.71

**Published:** 1995

**Authors:** Enrique Lehmann-Willenbrock, Kurt Semm, Jutta Lüttges, Lieselotte Mettler

**Affiliations:** Universitäts-Frauenklinik und Michaelis-Hebammenschule Kiel Michaelisstr. 16 Kiel D-24105 Germany

## Abstract

New abdominal and vaginal hysterectomy techniques, such as classic intrafascial serrated edged macro-morcellator (SEMM) hysterectomy (CISH), by pelviscopy/laparoscopy or laparotomy, and intrafascial vaginal hysterectomy (IVH), are both essentially supravaginal techniques. It has been claimed that they give a prophylaxis against cervical stump carcinoma by coring out the cervix with the SEMM. We set out to answer two questions: 1) How can vaginosonography help to choose an adequate SEMM diameter so that the cervical mucosa and transformation zone are completely removed, and 2) How often do cervical glands remain after the coring out procedure? We were able to show a good correlation between sonographic and histological morphology by giant and serial sections. In 253 CISH operations, resection of both endocervix and transformation zone was complete in 92.9%. Dysplasias were always removed completely; only 18 cervical cores exhibited healthy glands (retention cysts) in the resection margin. Therefore, CISH procedures should be able to prevent most of the cervical stump carcinomata that follow traditional supravaginal hysterectomy, but only long-term follow-up will give the final proof.


*Diagnostic and Therapeutic Endoscopy*, 1995, Vol. 2, pp. 71–77
Reprints available directly from the publisher
Photocopying permitted by license only

© 1995 Harwood Academic Publishers GmbH
Printed in Singapore

### Sonographic and Histological Morphometry of the Uterine Cervix—An Assessment of Laparoscopic and Other Intrafascial Hysterectomy Techniques

ENRIQUE LEHMANN-WILLENBROCK, KURT SEMM, JUTTA LÜTTGES and LIESELOTTE METTLER

*Universitäts-Frauenklinik und Michaelis-Hebammenschule Kiel, Kiel, Germany*

*(Received December 31, 1994; in final form, May 18, 1995)*

New abdominal and vaginal hysterectomy techniques, such as classic intrafascial serrated edged macro-morcellator (SEMM) hysterectomy (CISH), by pelviscopy/laparoscopy or laparotomy, and intrafascial vaginal hysterectomy (IVH), are both essentially supravaginal techniques. It has been claimed that they give a prophylaxis against cervical stump carcinoma by coring out the cervix with the SEMM. We set out to answer two questions: 1) How can vaginosonography help to choose an adequate SEMM diameter so that the cervical mucosa and transformation zone are completely removed, and 2) How often do cervical glands remain after the coring out procedure? We were able to show a good correlation between sonographic and histological morphology by giant and serial sections. In 253 CISH operations, resection of both endocervix and transformation zone was complete in 92.9%. Dysplasias were always removed completely; only 18 cervical cores exhibited healthy glands (retention cysts) in the resection margin. Therefore, CISH procedures should be able to prevent most of the cervical stump carcinomata that follow traditional supravaginal hysterectomy, but only long-term follow-up will give the final proof.

**KEY WORDS:** Hysterectomy methods, laparoscopy, pelviscopy, vaginosonography, cervix uteri, histomorphology, cervical cancer prophylaxis, cervical dysplasia

### INTRODUCTION

New hysterectomy techniques have been developed that claim to combine the minimal invasive approach of the supracervical method with the carcinoma prophylaxis of total hysterectomy (1,2). The procedures of classic intrafascial SEMM hysterectomy (CISH) and intrafascial vaginal hysterectomy (IVH) are suitable for this purpose, because a new instrument, called the serrated edged macro-morcellator (SEMM), removes the cervical mucosa while the pericervical tissue remains intact. The SEMM cores out the central part of the cervix and has a diameter of 10, 15, 20, or 24 mm. It is centered on the cervical canal with the aid of a previously placed guide rod and a circular distance holder where it functions as an integral part of the calibrated uterine resection tool (CURT). The cored-out tissue cylinder is suitable for precise histological evaluation, so that the resection margins may be examined as in cone biopsies.

Address for correspondence: Dr. med. E. Lehmann-Willenbrock, Universitäts-Frauenklinik und Michaelis-Hebammenschule Kiel, Michaelisstr. 16, D-24105 Kiel, Germany.

### MATERIALS AND METHODS

### Patient Selection

We included only patients with benign uterine disease requiring hysterectomy. To plan the adequate procedure, we performed a bimanual vaginal examination and wet preparation to rule out a vaginal infection. In patients with menorrhagia, dilatation and curettage was performed first to exclude endometrial carcinoma. We considered CISH only, if Papanicolaou smear and colposcopic findings were normal; otherwise, a cervical carcinoma had to be ruled out. All benign diseases of the uterus that could not be treated conservatively were considered as an indication for the procedure. Vaginosonography served to rule out malignancy and to take the measurements (see below) required to choose the adequate size of the CURT, i.e., 10, 15, 20, or 24 mm outer diameter. In some patients, we see ectropion such that its diameter exceeds the diameter of the cervix at its narrowest point. In these patients, a conization is performed as part of the procedure (see Fig. 7). In most patients this can be avoided if a large enough CURT is chosen.

### Sonographic and Histological Methods

First, we performed a detailed analysis of the first, consecutive 34 patients (ages: 30 to 62 years, averaging 41 years) who underwent the CISH procedure for benign uterine pathological changes. In 21 patients CISH was done by pelviscopy and in 13 patients by laparotomy.

To choose the adequate diameter of the CURT, bimanual palpation and vaginosonographic determination of the maximum diameter of the cervical mucosa were carried out. In the sonographic B picture, the mucosa is less echogenic than the surrounding fibromuscular tissue.

A sonographic distinction is not possible between the cervix and the isthmus uteri. Therefore, we measured the added length of both. The third parameter evaluated was the total diameter of the cervix (Fig. 1).

The cervix-cavum-fundus-tissue cylinder was embedded in paraffin. In eight cases, we prepared histological giant sections; the other 26 cylinders were prepared in serial cuts like a cone, with additional cross-sections in the isthmus region. Longitudinal sections (Fig. 2) and cross-sections (Fig. 3) were stained with hematoxylin-eosin. In all cases, we determined 1) the area of the cervical mucosa; 2) the length of the cervix; 3) the mucosal thickness at 0, 1, and 2 cm from the external os; and 4) the shortest distance between endocervical glands/retention cysts and the outer resection margin.

Having analyzed the first 34 patients thoroughly, we continued a less extensive analysis until the first 253 CISH patients from 1991 to 1993 had been investigated.

### RESULTS

### Sonographic and Histological Findings for the First 34 Patients

In all specimens, the cervical cavity was complete. In three patients, a mild squamous epithelial dysplasia (CIN I) was found in the transformation zone, and in three pa-

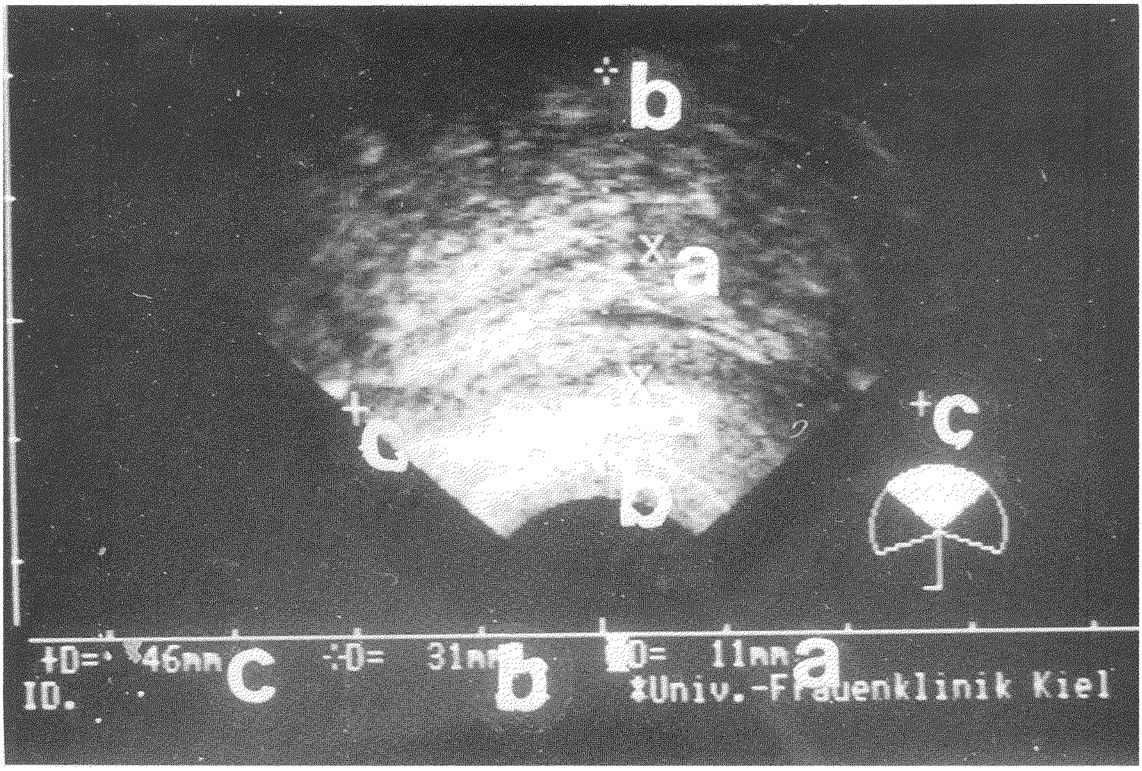

**Figure 1** Vaginosonographic picture of the uterine cervix. a-a, maximum diameter of cervical mucosa; b-b, added length of cervix and uterine isthmus; c-c, total diameter of cervix.

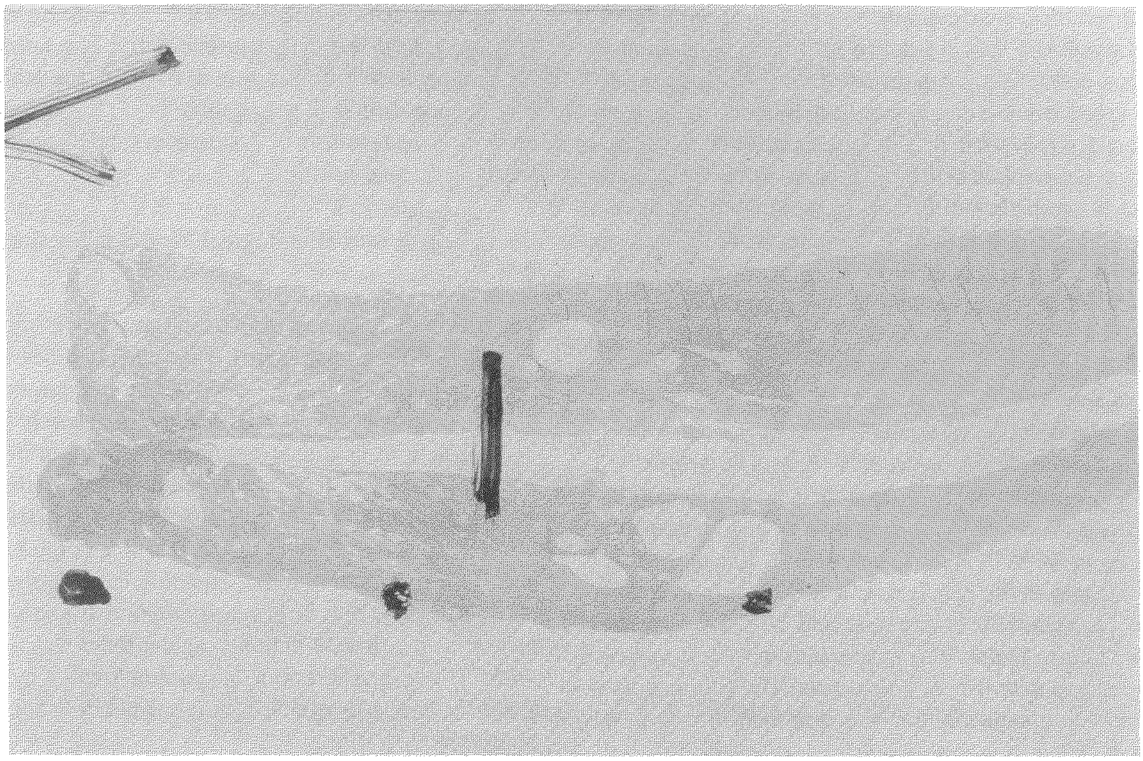

**Figure 2** Longitudinal section of cervical tissue cylinder. Observe retention cysts close to resection margin.

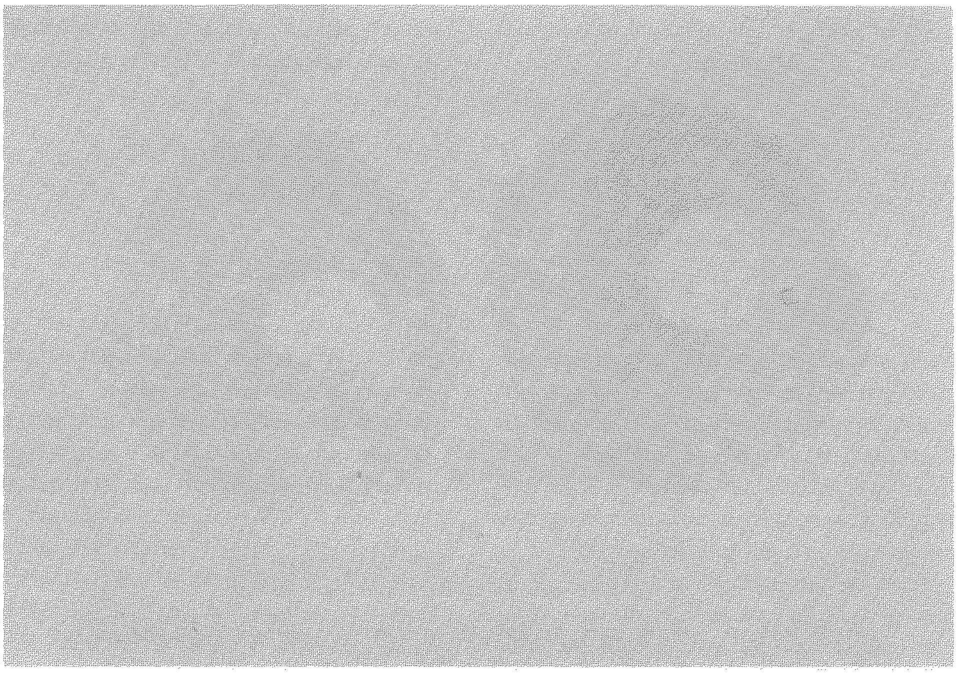

**Figure 3** Cross-section of cervical tissue cylinder. Observe broad fibromuscular margin around narrow band of cervical mucosa.

tients an endocervical squamous epithelial metaplasia without dysplasia was found. In all of the 34 patients, squamous epithelium without dysplasia was present at the outer resection border. In three patients, ectropion was discovered. Only one of the three patients required conization before the coring-out process to assure that all the glands from the outer cervix would be removed. Retention cysts up to 5 mm in diameter were present in nine patients; in seven of these, they were completely contained in the cylinder, and in two patients the cysts reached the outer resection margin. The epithelium that covered these cysts was flat and atrophic, without proliferative activity.

In the 34 patients investigated, there was no squamous epithelium in the deeper layers of the wall. The dysplasias were always in the transformation zone and had been removed in all cases. Morphometry showed an average mucosal area of 34 ± 19 $mm^2$. The mucosal thickness was 0.28 ± 0.2 mm at the ectocervix, 2.7 ± 1.7 mm at a distance of 1 cm from the external os, and 2.1 ± 1.2 mm at a distance of 2 cm. These data confirm the well-known spindle-shaped geometry of the endocervix. The shortest distance between glands and the resection margin was 1.99 ± 1.4 mm. From this, one would indeed expect to find glands reaching the resection margin in two patients. Vaginosonography showed a cervical length of 3.29 ± 0.54 cm, and morphometry a length of 2.84 ± 0.56 cm. The sample correlation between sonographic and morphometric lengths was $r = 0.74$; the probability that the correlation is not positive is < 0.1%. Linear regression gives the standard error for the estimation of the true cervix length from sonography of $s = 0.42$ cm.

The mucosal diameter was 9.23 ± 2.6 mm by sonography and 5.3 ± 3.4 mm by morphometry. The sample correlation between both values was $r = 0.54$; the probability that the correlation is not positive is < 1%. The standard error for the estimation of the endocervical thickness from sonography was 1.4 mm.

### Sonographic and Histological Findings for the First 253 Patients

The cervix diameter by vaginosonography was more than 2 cm in most patients (Fig. 4). This, and the increasing experience of the surgeons led to the fact that larger CURT diameters were chosen more and more frequently (Fig. 5). Still, a fraction of patients remained in whom cervix glands were present in the resection margins (Fig. 6). There were 15 dysplasias (9 CIN I, 4 CIN II, and 2 CIN III), which were removed with clear tissue margins (Fig. 7). There was no invasive carcinoma. Histological findings of the uterine corpus (Fig. 8) primarily demonstrated the same picture as the first 34 patients; again, there was no carcinoma. However, there was one patient, treated by laparotomy, in whom a leiomyosarcoma had not been anticipated before the operation.

### DISCUSSION

Malignancy in the Müllerian duct originates most often from the endothelium of the uterine cervix, especially the transformation zone, and the endometrium. Primary malignant degeneration of the cervical muscular and connec-

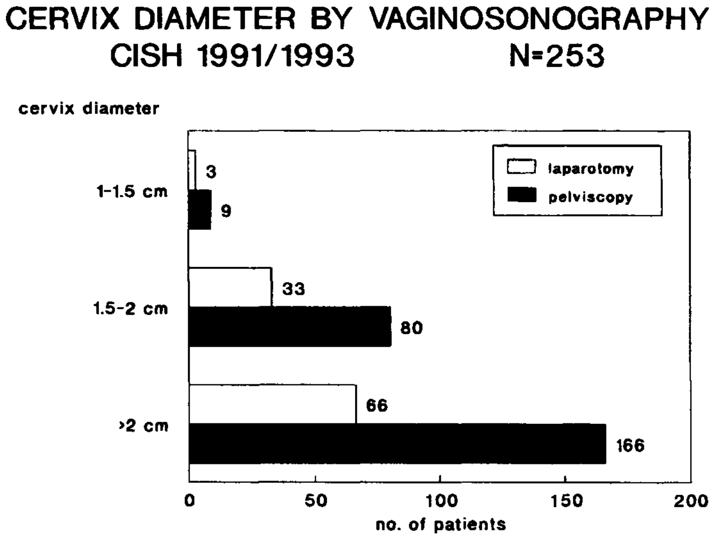

**Figure 4** Cervix diameter by vaginosonography.

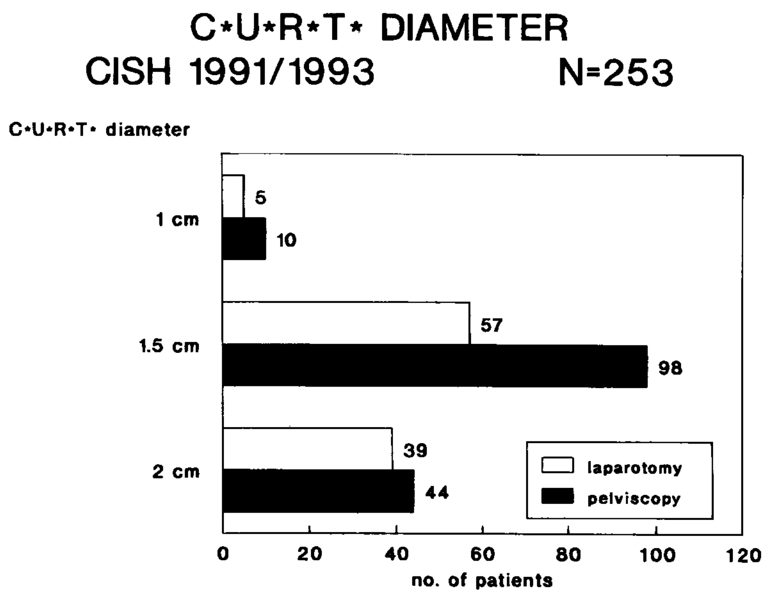

**Figure 5** CURT diameter.

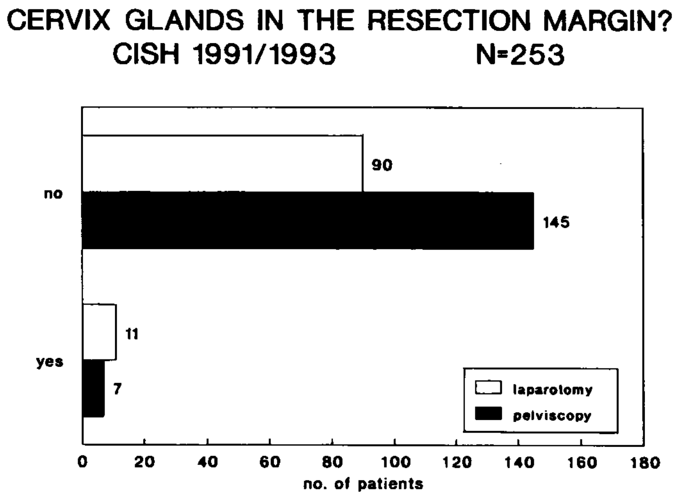

**Figure 6** Cervical glands in the resection margin.

tive tissue is virtually unknown, and carcinomata arising from the Gartner duct in the cervical wall are very rare (3).

Surgeons therefore began to look for a method of removing the cervical mucosa and the transformation zone when performing supracervical hysterectomy. However, the coagulation of the cervical canal (4) did not find much approval; postoperative curettage of the cervical stump showed that adenomatous epithelium was still present in approximately 66% of patients(5). Although they offered more security in the removal of the cervical mucosa, neither the transabdominal excision of the latter (6) nor the combination with transvaginal high-frequency current conization (7) became very popular because hemostasis remained a major problem. When the CISH procedure was developed, this aspect was thoroughly dealt with. With the described ligation technique for the cervical stump and endocoagulation with the WISAP Hemostaser and Erystop, we were able to obtain adequate intraoperative hemostasis. Both cervical length and mucosal diameter were larger in sonographic measurements, compared with morphometry. One of the reasons is postfixation and postembedding shrinkage of paraffin-embedded and stained tissue. Another factor is that the isthmus uteri and the cervical lumen are included in sonographic measurements. The

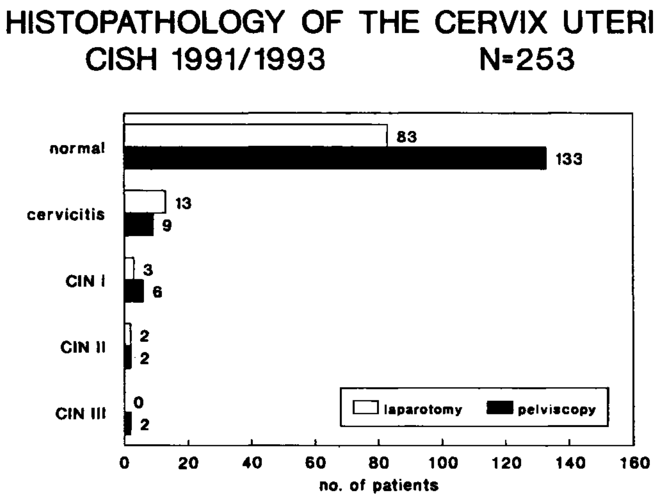

**Figure 7** Histopathological findings of the uterine cervix.

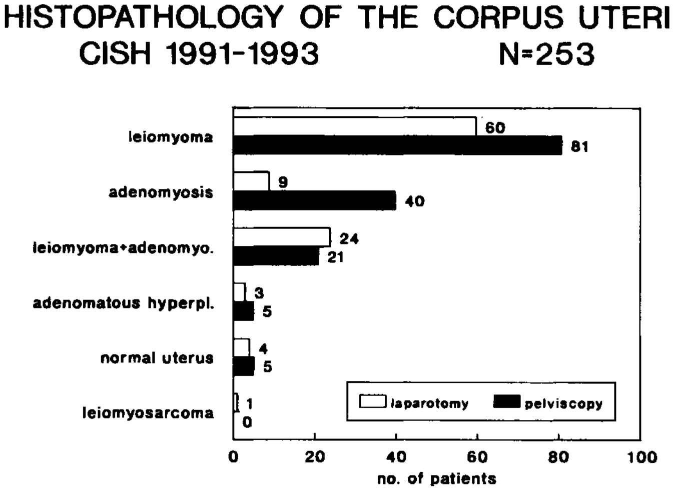

**Figure 8** Histopathological findings of the uterine corpus.

cervical lumen cannot be ascertained by morphometry, because there is an uncontrolled artificial distention of the two sides during the cutting procedure.

In two of the first 34 patients, retention cysts had not been removed completely. As there are no reports about carcinoma developing in the depth of retention cysts, this is not important; a second operation for the removal of these cysts is not necessary. Although more experience was gathered in the course of the study, the rate of incomplete removal of cervix glands remained virtually unchanged (16 of the next 219 patients). Therefore, 5 to 10% of the patients may have less protection against cervical stump carcinoma. On the other hand, coring out is followed by coagulation, which may well reduce that percentage. It should be kept in mind that even after simple high-frequency coagulation of the cervix (4), which leaves some mucosa behind in 66% of patients (8), the rate of cervical stump carcinoma is reduced to 0.11% (5). This compares rather favorably with the 0.3 to 1.8% rate following conventional supracervical hysterectomy.[9]

We may conclude:

1. Sonographic measurements allow a good estimation of cervical length and mucosal diameter. Thus, the resection tool for the mucosa can be chosen adequately in CISH procedures.
2. The transformation zone was usually cored out completely with the CURT; only one patient of 34 needed an additional conization. With this combination, it was always possible to remove the transformation zone.

3. Cervical glands/retention cysts remained in 5 to 10% of patients. Retention cysts probably have no significance for cervical stump carcinoma.

4. The CISH procedure is less invasive than total hysterectomy and still potentially avoids the risk of cervical stump carcinoma that is present after supracervical hysterectomy. However, only long-term follow-up will show how much cancer prevention is improved by C.I.S.H. and IVH over traditional techniques.

### REFERENCES

1. Semm K. Hysterektomie per laparotomiam oder per pelviskopiam. Ein neuer Weg ohne Kolpotomie durch C*A*S*H*. Geburtsh Frauenheilk 1991;51:996–1003.
2. Semm K. Intrafasciale vaginale Hysterektomie (IVH) mit und ohne pelviskopische Assistenz. Geburtsh Frauenheilk 1993;53:000–000.
3. Truskett I, Constable WC. Clear cell adenocarcinoma of the cervix and vaginal vault of mesonephric origin. Cancer 1968;21:249.
4. Rauramo M. On excision of the cervical canal in conjunction with uterus amputation. Acta Obstet Gynecol Scand 1949;28:381. Cited in Kilkku F. Grönroos M. Preoperative electrocoagulation of endocervical mucosa and later carcinoma of the cervical stump. Acta Obstet Gynecol Scand 1982;61:265–267.
5. Kilkku P. Grönroos M. Peroperative electrocoagulation of endocervical mucosa and later carcinoma of the cervical stump. Acta Obstet Gynecol Scand 1982;61:265–267.
6. Aldridge AHS, Meredith S. Am J Obstet Gynec 1950;59:748. Cited in Korte W. Die Bedeutung der Muskulatur des Collum uteri für die intraisthmische Technik der Uterusexstirpation, zugleich ein Beitrag zur Vermeidung des Kollumstumpf-Karzinoms. Geburtsh Frauenheilk 1964;24:211–218.
7. Ries J. Individuelle Indikation zur Art der Myomoperation. Arch Gynäkol 1961;195:225–227.
9. Kilkku F. Grönroos M. Taina E, et al. Colposcopic, cytological and histological evaluation of the cervical stump 3 years after supravaginal uterine amputation. Acta Obstet Gynecol Scand 1985;64:235–236.
10. Tervilä I. Carcinoma of the cervical stump. Acta Obstet Gynecol Scand 1963;42:200. Cited in Kilkku P. Grönroos M. Preoperative electrocoagulation of endocervical mucosa and later carcinoma of the cervical stump. Acta Obstet Gynecol Scand 1982;61:265–267.

